# New finite-size correction for local alignment score distributions

**DOI:** 10.1186/1756-0500-5-286

**Published:** 2012-06-12

**Authors:** Yonil Park, Sergey Sheetlin, Ning Ma, Thomas L Madden, John L Spouge

**Affiliations:** 1 National Center for Biotechnology Information, National Library of Medicine, Bethesda, MD, 20894, USA

## Abstract

**Background:**

Local alignment programs often calculate the probability that a match occurred by chance. The calculation of this probability may require a “finite-size” correction to the lengths of the sequences, as an alignment that starts near the end of either sequence may run out of sequence before achieving a significant score.

**Findings:**

We present an improved finite-size correction that considers the distribution of sequence lengths rather than simply the corresponding means. This approach improves sensitivity and avoids substituting an *ad hoc* length for short sequences that can underestimate the significance of a match. We use a test set derived from ASTRAL to show improved ROC scores, especially for shorter sequences.

**Conclusions:**

The new finite-size correction improves the calculation of probabilities for a local alignment. It is now used in the BLAST+ package and at the NCBI BLAST web site ( http://blast.ncbi.nlm.nih.gov).

## Background

Local alignments are an essential tool for biologists and often provide the first information about the function of an unknown nucleotide or protein sequence. An important question concerns the relationship of the score of a local alignment with the probability that the alignment occurred by chance. [Karlin and Altschul 1] developed an asymptotic theory for local alignments, assuming that no gaps are permitted. For two random sequences **I** and **J** of lengths *m* and *n*, respectively, the resulting distribution of the optimal alignment score $\hat{M}$ approximates a Gumbel distribution [2]

(1) $$P\left\{\hat{M}>y\right\}\approx 1-\exp\left(-kmne^{-\lambda y}\right)\text{.}$$

The two statistical parameters in Equation (1) are *λ*, the scale parameter, and *k*, the pre-factor.

Several authors [3-12] extended this framework to local alignments with gaps and showed that the Gumbel distribution from Equation (1) is still valid, though different values for *λ* and *k* are required. [Altschul 13] discussed the need for a “finite-size correction” to the lengths *m* and *n* to improve the accuracy of Equation (1). The resulting statistics are an integral part of the Basic Local Alignment Search Tool (BLAST) [14].

**Figure 1:**
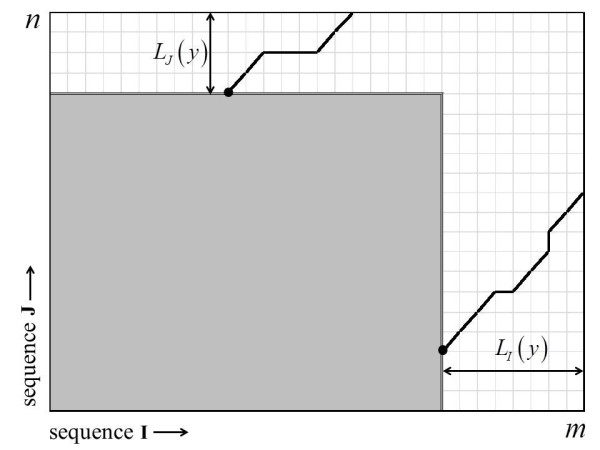
**Sequence alignment graph of two random sequences I and J of lengths *m *and *n*, respectively.** The black circles are the initiation vertices of local alignment paths just remaining within the large rectangle of the sequence alignment graph before achieving the score *y*, i.e., the lower local alignment path has length *L*_*I*_ (*y*) within **I**; and the upper, length *L*_*J*_ (*y*) within **J**. The gray shaded area is therefore the (random) alignment rectangle that an optimal local alignment must start within to achieve the score *y*. Thus, if the black circles lie within the gray rectangle, the alignments achieve the score *y* within *m* × *n* alignment rectangle. If the black circles lie further outside the gray rectangle, the alignments do not achieve the score *y* within *m* × *n* alignment rectangle.

The following presentation emphasizes intuition over mathematical formality, to explain how the finite-size correction can account for the finite sequence lengths *m* and *n* to improve the accuracy of Equation (1). Let us begin with an optimal local alignment, which starts from score 0 and requires a non-zero sequence length within both **I** and **J**, before it achieves score *y*. Let *L*_*I*_ (*y*) (*L*_*J*_ (*y*)) be the required random lengths within both **I** (**J**), and let $l_{I}\left(y\right)=E\left\{L_{I}\left(y\right)\right\}$ ( $l_{J}\left(y\right)=E\left\{L_{J}\left(y\right)\right\}$) be the corresponding means. The main idea is that the optimal local alignment cannot start anywhere along the full length *m* (*n*) of sequence **I** (**J**), because there might be insufficient sequence to permit it to achieve the score *y* (Figure 1). The finite-size correction described in [13] and used in BLAST therefore replaced the area *mn* of the alignment matrix for Equation (1) by

(2) $$\left[m-l_{I}\left(y\right)\right]\left[n-l_{J}\left(y\right)\right]\text{.}$$

Equation (2) approximates the area within the alignment matrix where the optimal local alignment can start and on average still have enough space to exceed the score *y*. If *m* < *l*_*I*_ (*y*) or *n* < *l*_*J*_ (*y*), however, the resulting value in Equation (2) might become negative. The BLAST code for the old finite-size correction therefore set the corrected sequence length to an *ad hoc* value (typically 1). For very short query or database sequences, the *ad hoc* correction could underestimate the significance of an alignment by many orders of magnitude.

The purpose of this note is to present a new finite-size correction formula for the BLAST statistics. It avoids the *ad hoc* correction and improves on them by considering the (approximately normal) distributions of the random lengths *L*_*I*_ (*y*) and *L*_*J*_ (*y*) explicitly, and not just the corresponding means *l*_*I*_ (*y*) and *l*_*J*_ (*y*). We demonstrate below that the new finite-size correction is better than the older one, both in theory and in practice. All BLAST+ protein-protein applications (i.e., BLASTP, BLASTX) use the new finite-size correction by default, starting with version 2.2.26.

## Findings

### New finite-size correction

As with the old finite-size correction, the expectation $l_{I}\left(y\right)=EL_{I}\left(y\right)$ is approximated linearly:

(3) $$l_{I}\left(y\right)=a_{I}y+b_{I}\text{.}$$

Most practical scoring systems are symmetric, with *s*(*A*, *B*) = *s*(*B*, *A*) for any two letters *A* and *B*, and for a symmetric scoring matrix and symmetric sequence compositions, expectations corresponding to **I** and **J** are the same, e.g., *l*_*I*_ (*y*) = *l*_*J*_ (*y*) = *l*(*y*). For asymmetric scoring systems or asymmetric sequence compositions, however, the variates *L*_*I*_ (*y*) and *L*_*J*_ (*y*) can have different distributions, so the following retains the subscripts **I** and **J**.

The new finite-size correction replaces *mn* in Equation (1) by

(4) $$E\left\{{\left[m-L_{I}\left(y\right)\right]}^{+}{\left[n-L_{J}\left(y\right)\right]}^{+}\right\}\text{,}$$

where x^+^ = max{*x*,0}. Rather than taking the expectation of*L*_*I*_ (*y*) and *L*_*J*_ (*y*) as in Equation (2), Equation (4) is the expected area within the alignment rectangle where an optimal local alignment can start and have enough random sequence length to reach the score *y* (Figure 1).

The practical computation of Equation (4) approximates the distribution of (*L*_*I*_(*y*),*L*_*J*_ (*y*)) with a bivariate normal distribution, with means $l_{I}\left(y\right)=EL_{I}\left(y\right)$and $l_{J}\left(y\right)=EL_{J}\left(y\right)$,variances var *L*_*I*_ (*y*) = *v*_*I*_ (*y*) and var *L*_*J*_ (*y*) = *v*_*J*_ (*y*), and covariance cov (*L*_*I*_(*y*), *L*_*J*_ (*y*)) = *c*(*y*), all assumed to be linear in the score *y*, i.e.,

(5) $$\begin{array}{c} l_{I}\left(y\right)=a_{I}y+b_{I},l_{J}\left(y\right)=a_{J}y+b_{J}\text{,}\;\\ \;v_{I}\left(y\right)=\alpha_{I}y+\beta_{I},v_{J}\left(y\right)=\alpha_{J}y+\beta_{J},\\ \;c\left(y\right)=\sigma y+\tau \text{.} \end{array}$$

The estimation of the parameters *a*_*I*_*a*_*J*_*α*_*I*_*α*_*J*_ and *σ* has mathematical depth and involves many unproved speculations, but involves a heuristic modeling of a random sequence alignment with Markov additive processes [15], ultimately with use of the renewal-reward theorem. The Appendix presents formulas for computing *a*_*I*_*a*_*J*_*α*_*I*_*α*_*J*_ and *σ*.

BLAST *p*-values are relatively insensitive to the values of the intercepts *b*_*I*_*b*_*J*_*β*_*I*_*β*_*J*_, and *τ*, so the practical computation approximates them, as follows. Let *a*_*u*_ (*α*_*u*_) be the value of *a*_*I*_ (*α*_*I*_) for ungapped alignment. The mathematical theories for random walks and for renewals yield analytic formulas for *a*_*u*_ and *α*_*u*_[16]. For an ungapped optimal alignment, the alignment length required to exceed the score *y* is the same within the sequences **I** and **J**, because it lacks gaps. Thus, *a*_*u*_ and *α*_*u*_ do not depend on the sequence (**I** or **J**) under consideration, so they contain no subscripts **I** or **J**. In a gapped alignment, let a gap of length 1 incurs a penalty *G*. The following uncontrolled approximations hold [17]:

(6) $$\begin{array}{c} b_{I}=2G\left(a_{u}-a_{I}\right),b_{J}=2G\left(a_{u}-a_{J}\right)\\ \beta_{I}=2G\left(\alpha_{u}-\alpha_{I}\right),\beta_{J}=2G\left(\alpha_{u}-\alpha_{J}\right)\\ \tau =2G\left(\alpha_{u}-\sigma \right)\text{.} \end{array}$$

Under the normal approximation, routine computation shows that Equation (4) is approximately

(7) $$\begin{array}{l} E\left({\left[m-L_{I}\left(y\right)\right]}^{+}{\left[n-L_{J}\left(y\right)\right]}^{+}\right)\approx \\ \left[\left(m-l_{I}\left(y\right)\right)P\left(X\leq m_{\Phi }\right)-\sqrt{v_{I}\left(y\right)}E\left(X;X\leq m_{\Phi }\right)\right]\times \\ \left[\left(n-l_{J}\left(y\right)\right)P\left(X\leq n_{\Phi }\right)-\sqrt{v_{J}\left(y\right)}E\left(X;X\leq n_{\Phi }\right)\right]+\\ c\left(y\right)P\left(X\leq m_{\Phi }\right)P\left(X\leq n_{\Phi }\right) \end{array}\text{,}$$

where $m_{\Phi }:=\left[m-l_{I}\left(y\right)\right]/\sqrt{v_{I}\left(y\right)}$, $n_{\Phi }:=\left(n-l_{J}\left(y\right)\right)/\sqrt{v_{J}\left(y\right)}$, and *X* is a standard normal variate. The final product $P\left(X\leq m_{\Phi }\right)P\left(X\leq n_{\Phi }\right)$ is an uncontrolled independence approximation for the bivariate normal distribution.

### Comparison of *p*-values for the new and old finite-size corrections

We compared *p*-values for the new finite-size correction with those for the old finite-size correction using the BLOSUM62 scoring matrix and affine gap penalty 11 + *g*. Hartmann used a rare-event simulation method to compute the local alignment score distribution for ranges that included small *p*-values like *p* = 10^−50^[18], thereby producing a theoretical standard for small *p*-values.

**Figure 2:**
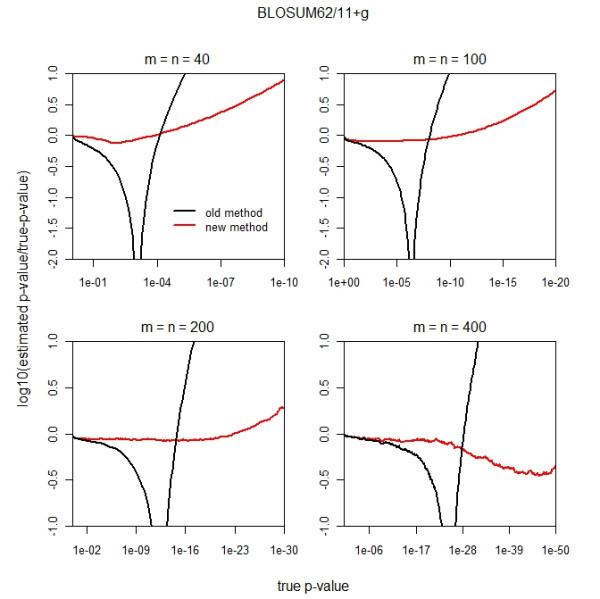
**Comparison of *p*-values for the new and old finite-size corrections using the BLOSUM62 scoring matrix and 11 + *g *affine gap penalty for equal sequence lengths (*m*=*n*) 40, 100, 200, and 400.** Figure 2 plots $\log_{{}_{10}}\hat{p}/p$ against *p*, where $\hat{p}$ is the calculated *p*-value and *p* is the *p*-value from the simulation. Thus, a perfect *p*-value estimate corresponds to the horizontal line *y* = 0. The red line shows the results from the new finite-size correction; the black line, the old finite-size correction.

Figure 2 plots relative errors in logarithmic scale against true *p*-values for equal sequence lengths *m* = *n* = 40, 100, 200, and 400. Using Hartmann’s theoretical standard, the new finite-size correction outperforms as the *p*-value decreases, sometimes by orders of magnitude.

### Evaluation of accuracy

We evaluated the performance of the new finite-size correction using the ASTRAL SCOP 40 subset [19] of release 1.75 of the Structural Classification of Proteins (SCOP) [20] database. We sorted the SCOP domains by lexicographic order and used the even numbered sequences as our query set, but removed any query that was the sole member of the superfamily in ASTRAL 40. For a given query sequence, we considered any database sequence belonging to the same SCOP superfamily as a true positive, and any database sequence belonging to a different SCOP fold as a false positive. Following [21], in the retrieval list for each query, we censored all sequences belonging to the same fold but different superfamily, so those sequences contributed neither true or false positives to the retrieval.

We report the performance in terms of the Receiver Operator Characteristics (ROC). Specifically, we report the ROC_*n*_ score, which is obtained by pooling the results of all queries, ordering them by expect value, but only keeping results up the *n*-th false positive [21]. The expect value for the database search was obtained from the pairwise *p*-values using a length-proportional correction that takes the ratio of the database length to the target sequence length into account [13].

As discussed above, the new finite-size correction should show the greatest improvement for short sequences. Therefore, we also produced ROC_*n*_ scores for different subsets of the SCOP database. One database subset has sequences shorter than the 25th percentile length (95 residues), and another has sequences shorter than the 50th percentile length (137 residues).

**Figure 3:**
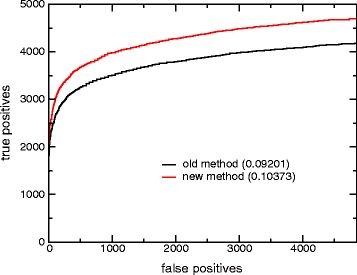
**Number of true positives vs. number of false positives for both new and old finite-size corrections using short SCOP sequences as a database.** The searched database was created from the shortest 25% of the ASTRAL 40 sequences for SCOP version 1.75 (see text).

Table 1 presents ROC_*n*_ scores for the full database as well as the two subsets described above. These scores have an average of one false positive per query (4852), a threshold found useful in other studies (Altschul SF, private communication). The ROC-4852 scores for the full database demonstrate a small improvement of the new finite-size correction over the older one. The subsets show a more impressive improvement. For the 50^th^ percentile subset, the ROC-4852 score improves by 9%. For the 25^th^ percentile subset, the ROC-4852 score shows a 13% improvement. In the 25^th^ percentile subset, the new finite-size correction produces roughly 12% more true positives overall at 4852 false positives than the old finite-size correction (Figure 3). These results confirm our expectation that the new finite-size correction will display greatest improvement in retrieval for short sequences.

**Table 1:** Retrieval accuracy for different subsets of SCOP database with the new and old finite-size correction

**Method**	**25^th^ percentile**	**50^th^ percentile**	**Full database**
New correction	0.10373 ± 0.00022	0.10073 ± 0.00019	0.08535 ± 0.00013
Old correction	0.09201 ± 0.00020	0.09282 ± 0.00017	0.08358 ± 0.00014

The three subsets contain proteins shorter than 91 residues (25^th^ percentile by length), shorter than 137 residues (50^th^ percentile by length), and the full database. ROC-4852 scores are presented with an error (one standard deviation). The 25^th^ percentile database contains 2533 sequences, the 50^th^ percentile database contains 5008 sequences, and the full database contains 10,569 sequences. There are 4852 queries.

To assess the significance of this improvement on BLAST searches, one may look to the length distribution of sequences in a heavily used protein BLAST database. The non-redundant (“nr”) database is the default protein database at the NCBI BLAST web site. Of the sequences in the nr database, 11% are 95 residues or shorter; and 21%, 137 residues or shorter. The new finite-size correction improves the retrieval accuracy for a noticeable fraction of the proteins in the nr database.

## Conclusion

We have described a new finite-size correction. The new correction has a more rigorous derivation than the current finite-size correction and avoids the use of an *ad hoc* value for short sequences. We have tested the retrieval accuracy of the new finite-size correction on the gold standard SCOP set, and have shown that the improvement is most important for short sequences. This correction has been made part of the BLAST+ protein-protein applications (e.g., BLASTP, BLASTX) as well as at the NCBI BLAST web site. In the future, we plan to implement this correction for nucleotide-nucleotide comparisons.

## Availability and requirements

Project Name: BLAST Statistical Parameters

Project home page: http://www.ncbi.nlm.nih.gov/CBBresearch/Spouge/html_ncbi/html/blast/

Operating systems: Windows, MacOSX, LINUX, UNIX

Programming language: C++

License: Public Domain (see http://www.ncbi.nlm.nih.gov/books/NBK22952/)

Any restrictions to use by non-academics: None

## Competing interests

The authors declare that they have no competing interests.

## Authors’ contributions

YP, TM and JS drafted the manuscript. YP designed the *p*-value evaluation method. SS implemented the new finite-size correction. NM integrated the correction into the BLAST+ code, ran tests, and calculated the ROC scores. JS devised the new finite-size correction. YP and SS are equal contribution first authors for this article. TLM and JLS are equal contribution last authors for this article. All authors read and approved the final manuscript.

## Appendix

Let $Z_{+}=\{0,1,\ldots \}$. Consider two semi-infinite random sequences, yielding alignment scores *S*_*i,j*_ at each vertex $\left(i,j\right)\in Z_{+}^{2}$ within their alignment graph. Define the edge maximum score $E_{n}=\max\left\{\max_{0\leq i\leq n}S_{i,n},\max_{0\leq j\leq n}S_{n,j}\right\}$. Let $\kappa_{0}=E_{\kappa_{0}}=0$ and $\kappa_{i}=\inf\left\{n:n>\kappa_{i-1},E_{n}>E_{\kappa_{i-1}}\right\}$ for *i* ≥ 1. We call *κ*_*i*_ the *i*^*th*^ SALE (strict ascending ladder epoch) and $E_{\kappa_{i}}$ the *i*^*th*^ SALE score. Let $\Delta E_{i}^{}:=E_{\kappa_{i}}^{}-E_{\kappa_{i-1}}^{}$, the increment between the (*i* − 1)^*th*^ and *i*^*th*^ SALE scores.

Let $L_{I}\left(y\right)=\inf\left\{i:S_{i,j}\geq y,\left(i,j\right)\in Z_{+}^{2}\right\}$ and $L_{J}\left(y\right)=\inf\left\{j:S_{i,j}\geq y,\left(i,j\right)\in Z_{+}^{2}\right\}$. We also define $I_{n}=\inf\left\{i:S_{i,j}=E_{\kappa_{n}},\left(i,j\right)\in Z_{+}^{2}\right\}$and $J_{n}=\inf\left\{j:S_{i,j}=E_{\kappa_{n}},\left(i,j\right)\in Z_{+}^{2}\right\}$. Let Δ*I*_*i*_ := *I*_*i*_ − *I*_*i*−1_, the incremental sequence length between (*i* − 1)^*th*^ and *i*^*th*^ SALEs in sequence **I**, and Δ*J*_*j*_ := *J*_*j*_ − *J*_*j*−1_, the incremental sequence length between (*j* − 1)^*th*^ and *j*^*th*^ SALEs in sequence **J**. Last, we define $E^{*}\left[\Delta I_{i}\right]:=E\left[\Delta I_{i}e^{\lambda E_{\kappa_{i}}},\kappa_{i}<\infty \right]$, $E^{*}\left[\Delta J_{j}\right]:=E\left[\Delta J_{j}e^{\lambda E_{\kappa_{j}}},\kappa_{j}<\infty \right]$, and $E^{*}\left[\Delta E_{i}\right]:=E\left[\Delta E_{i}e^{\lambda E_{\kappa_{i}}},\kappa_{i}<\infty \right]$.

The formulas for computing *a*_*I*_*a*_*J*_*α*_*I*_*α*_*J*_ and *σ* are:

(8) $$\begin{array}{l} a_{I}=\lim_{i\to \infty }\frac{E^{*}\left[\Delta I_{i}\right]}{E^{*}\left[\Delta E_{i}\right]},a_{J}=\lim_{j\to \infty }\frac{E^{*}\left[\Delta J_{j}\right]}{E^{*}\left[\Delta E_{j}\right]},\\ \alpha_{I}=\lim_{i\to \infty }\frac{{\mathrm{var}}^{*}\left[\Delta I_{i}\right]}{E^{*}\left[\Delta E_{i}\right]},\alpha_{J}=\lim_{j\to \infty }\frac{{\mathrm{var}}^{*}\left[\Delta I_{j}\right]}{E^{*}\left[\Delta E_{j}\right]},\\ \sigma =\lim_{i\to \infty }\frac{{\mathrm{cov}}^{*}\left[\Delta I_{i},\Delta J_{i}\right]}{E^{*}\left[\Delta E_{i}\right]}, \end{array}$$

where var^*^ and cov^*^ represent the variance and covariance associated with the probability measure underlying the expectation $E^{*}$. In practice, for computational efficiency, we use importance sampling to estimate the parameters above [15]. The parameters are estimated separately at each SALE. We then apply asymptotic regression to estimate the values of *a*_*I*_*a*_*J*_*α*_*I*_*α*_*J*_ and *σ* as *i* → ∞ in the equation above [22].
